# The Dark Value of Criminal Bodies: Context, Consent, and the Disturbing Sale of John Parker’s Skull

**DOI:** 10.5334/jcms.1021220

**Published:** 2015-02-09

**Authors:** Shane McCorristine

**Affiliations:** * Wellcome Trust Postdoctoral Fellow University of Leicester, UK

**Keywords:** Criminal body, display, execution, auction, organ theft

## Abstract

The recent sale of a human skull at an auction in Sussex should raise ethical concerns. Whenever human body parts are sold or put in a glass case and displayed for public view people should be provided with context and extensively informed about what they see. The gaze is never innocent, and to ignore the particular journeys that body parts take into auction rooms, anatomy departments, and museums is to be complicit in acts of historical injustice. In this case the skull was that of John Parker, who was executed by hanging in 1813. The likelihood that this was illicitly obtained by medical professionals means that the sale of the skull at auction two hundred years later is fraught with ethical problems. Along with a discussion of context, fragments like Parker’s skull must therefore also become part of a debate about consent. Issues of context and consent can help us think about the display of human remains in museums in a manner that is intimate and ‘disturbingly informative’ (Mütter Museum 2014). However, the sale of Parker’s skull – described as an ‘antique piece’ in the press coverage (BBC News 2014) – is a reminder that the global marketplace in objectified body parts is disturbing in quite a different manner.^1^

## Introduction

**Figure 1:**
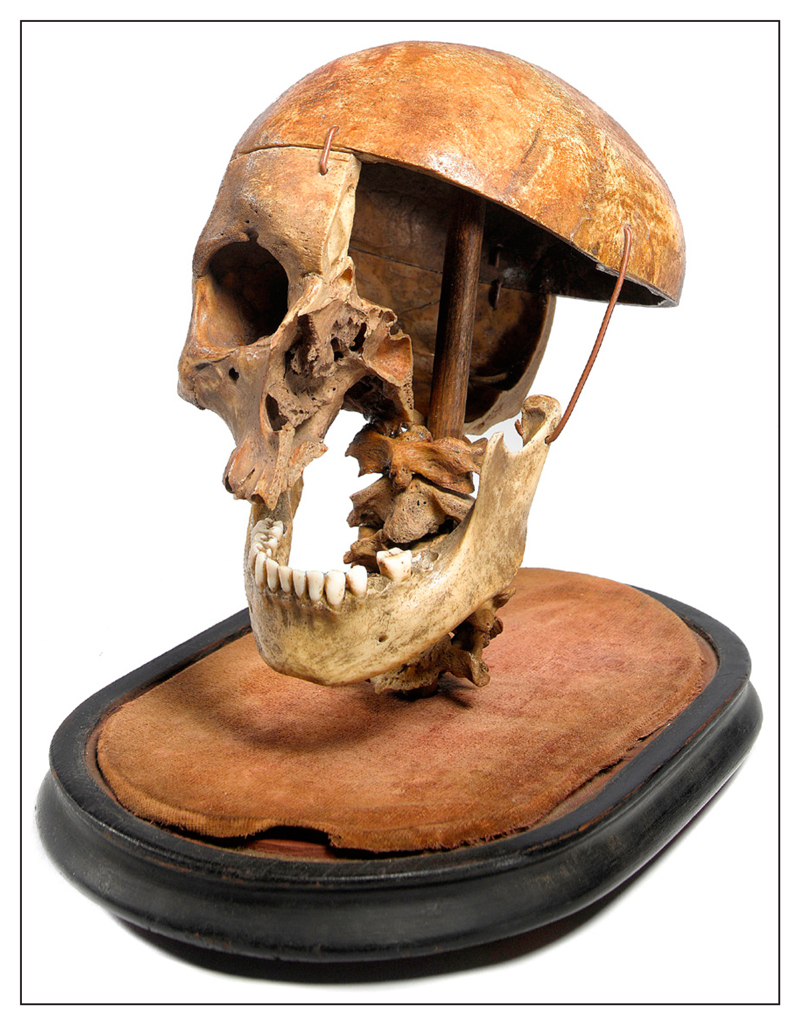
Parker’s skull (© Summers Place Auctions).

In May 2014 it was reported that the skull of John Parker, a 36-year-old man executed at Gloucester gaol in 1813, was sold at an auction in Sussex for £2,000. Described as an ‘antique piece’, the skull, displayed in a glass dome, was inscribed with the words, ‘John Parker hanged for robbing Henbury Church and De Boudrie’s school’ (Figure 1). The skull is 12-inches high and at some point has been partially cut away to serve as a teaching tool for anatomists. A photocopied newspaper cutting placed alongside the skull gave the barest historical details about the man, described as a ‘Wiltshire thief’. The auctioneer involved said ‘It is one of the more wacky items we have had’ (BBC News 2014).

How appropriate is it to think of Parker’s skull as a curiosity, as a wacky object that can be sold so casually? Is it wrong for the participants in this sale to leave unquestioned their attraction to the body parts of the criminal dead? This sale was disturbing for a number of reasons, and it should raise ethical concerns among those who sell, buy, and curate the body parts of people who have been executed. Skulls, in particular, are ‘good to think with’ in this regard because in many ways they are people that once were, they are ‘man’s final claim to individuality’ (Weil 1992: 272). Skulls house the brain and sensory systems, denote the physical appearance of a person, and symbolically represent the being and personalities of their owners (Quigley 2001: 5). There is of course a long tradition of skulls inspiring living people to contemplate mortality, vanity, and the impermanence of life – Hamlet’s monologue with the skull of poor Yorick is a good example of this (Frye 1979). Given the contemporary configurations of this practice of *Memento mori* – with DNA harvesting, organ donation, and cryogenics refashioning what it means to die – it is therefore imperative that pressing issues of context and consent be raised when items such as Parker’s skull come up for sale.

Whenever human body parts are put in a glass case and displayed for public view people should be provided with context and extensively informed about what they see. The gaze is never innocent and human remains acquire new meanings as they pass through the hands of different practitioners, custodians, and collectors (Alberti 2011). Therefore, ignoring the particular journeys that relics take into auction rooms, anatomy departments, death displays, and museums is methodologically unsound and acquiesces to acts of historical injustice.

## ‘Some further Terror and peculiar mark of Infamy’

Parker was executed during a period of a harsh system of law in England, known as the ‘Bloody Code’, which meant that hundreds of offences attracted the death penalty. Parker and his accomplice Thomas Rodway were unlucky in that the value of the items they stole allowed the judge to pass a sentence of death upon them. Rodway’s wife, it is worth mentioning, reportedly cut her throat in front of her children after her last interview with her husband (Hampshire Chronicle 1813).

Although they were recidivist thieves, Parker and Rodway were also victims of a structurally unequal judicial system which executed over 100 other men and women in 1813, mostly for non-lethal crimes. Indeed, in a notorious case of 1816 known as the ‘granny hanging’ (Mail on Sunday 2010), the 69-year-old Dinah Riddiford was executed in Gloucester for participating in her family’s theft of bacon, butter, and a copper kettle to the value of *£*4. Riddiford’s husband and son were acquitted of this offence, while another son had his death sentence commuted to transportation. Dinah Riddiford confessed her guilt and was promptly executed.

Riddiford (incidentally, an ancestor of Kylie Minogue) was allowed the dignity of a decent burial in Thornbury after her death, but this was something that was denied on principle to another category of criminals. The Murder Act of 1752 stipulated that all criminals executed for murder would not be buried, but either hung in chains (gibbeting) or dissected and anatomized. These post-mortem punishments were designed to act as a ‘further Terror and peculiar mark of Infamy’. Like gibbeting, the sentence of dissection and anatomization was intended to punish the criminal with the knowledge of his or her post-mortem fate while they were alive.

**Figure 2:**
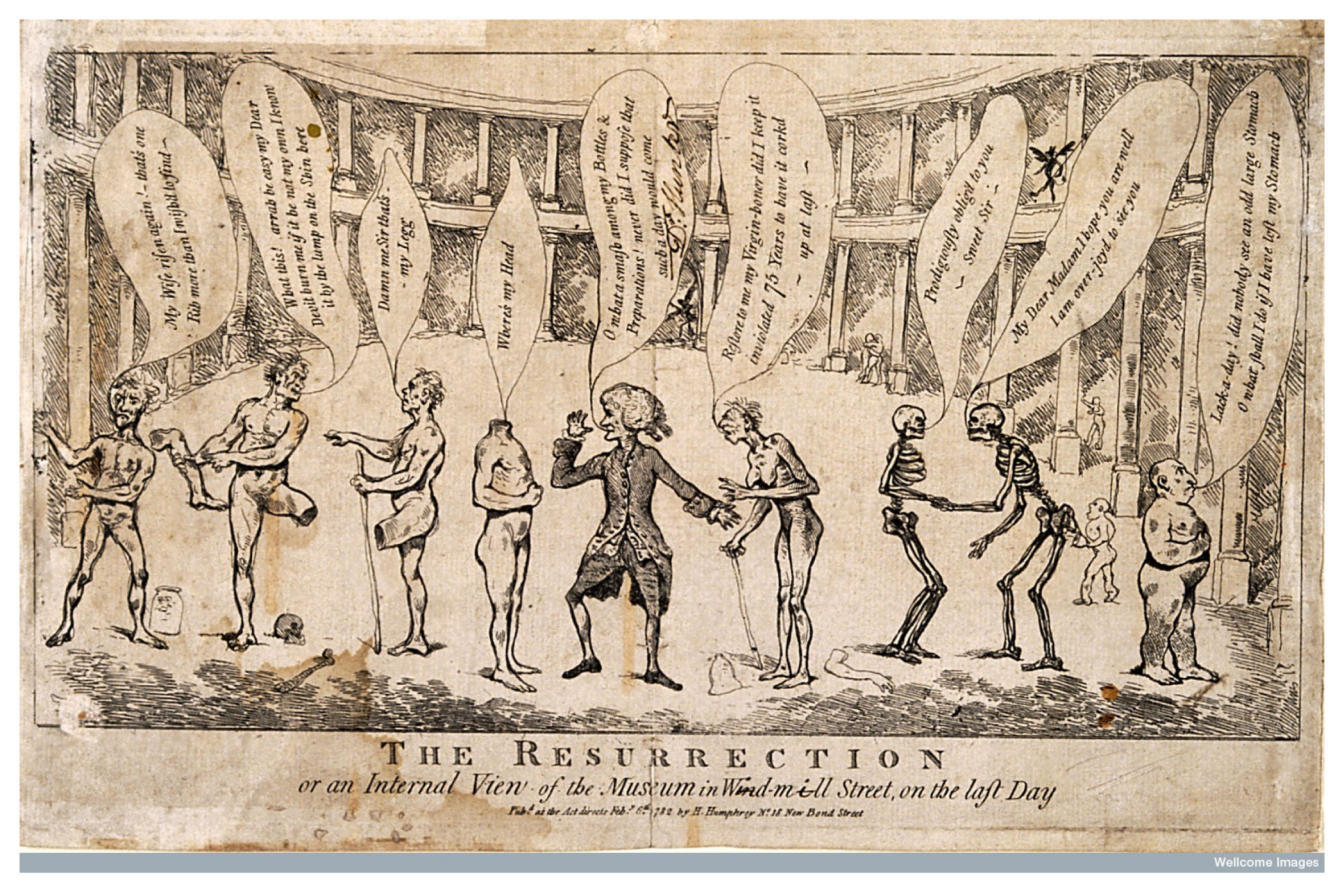
William Hunter (1718–1783) in his museum in Windmill Street on the day of resurrection, surrounded by skeletons and bodies, some of whom are searching for their missing parts. Engraving, 1782 (© Wellcome Images).

The context of this punishment was that the dismemberment of the body was seen as a ‘bad’ death, something that struck at commonly held beliefs about a decent Christian burial and the resurrection of the body whole (Tarlow 2010: 33–38). The result of this punishment was that medicine and medical museums, by using the bodies of criminals as objects of study, became complicit in the commodification and fragmentation of human remains (Figure 2). During the eighteenth century the scaffold at Tyburn occasionally became a site of riotous disorder as surgeons moved in to claim the bodies of executed murderers before their friends or families could cut them down and whisk them away for burial. This raises the question of why Parker’s corpse ended up as an anatomical specimen.

Although consent was something appropriated by the state, Parker was executed for theft and so his body should have been safe from the surgeon’s table then, and the catalogue of the auction house today. However, the journey of Parker’s skull from his death sentence in 1813 to Lot Number 202 in 2014 discloses the sophisticated illegal trade in corpses which tied together the judicial system, the medical establishment, and the ‘resurrectionists’ or body snatchers – people who procured the bodies of the recently dead (or in some cases murdered) for the burgeoning medical schools of England and Scotland. Although it was not unheard of, it is unlikely that Parker voluntarily sold his body to the surgeons. Therefore, it is most likely that someone stole or purchased Parker’s corpse and dismembered it, setting it on a journey of economic exchanges in which it became decontextualized, and this should not be forgotten. In fact, it is worth thinking about the effect of such a process:

‘Bodysnatching negated traditional death culture, tormented the dying, and denied the bereaved imaginative comfort. It nullified their efforts to ensure peaceful rest for the dead. It deprived the dead of their rightful place in the grave, and the future prepared for them, and it deprived survivors the comfort of knowing where to commune with them. Grave-robbery mangled grief processes by compounding these deprivations with the ominous threat of haunting, and ruptured hopes of companionable resurrection’ (Richardson 2006: 156).

## The Commodification of Dead Bodies

So far I have provided some context for the journey of Parker’s skull – the kind of information that might make it onto interpretive text panels in museums displaying human remains – but we can go further in making clear the threads that link Parker’s afterlife with the current journeys of other ‘divisible bodies’ (Scheper-Hughes 2001) in the global economy in human remains. It is not clear if Parker’s body underwent the same rituals of display, anatomisation, and fragmentation that were typically carried out on the bodies of executed murderers in the nineteenth century, but, given the numbers of medical students and shortage of corpses at the time, it is extremely likely. As the historians Ruth Richardson (1987) and Elizabeth Hurren (2012) have shown, nineteenth-century medical training encouraged respectable doctors to act in a way that would now be seen as utterly criminal. Body theft, sham funerals, and the targeting of the poorest and most vulnerable bodies in society mean that no amount of disinfectant can create a clinical distance between the trade in human remains 200 years ago and the organ and body trades of today. Then, as now, consent is not a simple thing and, without a rich contextual approach to the matter, we run the risk of passing over the structural inequalities that make it more or less likely for a person to end up in fragments, to literally ‘rest in pieces’. The skulls of certain types of people were at particular risk of theft and mobility from so-called ‘craniokleptics’ (Dickey 2009), especially indigenous peoples, executed criminals, creative geniuses, and those who died of rare diseases or conditions. Indeed, imperial ‘headhunting’ and the movement of subaltern skulls through Western museums and collections is currently a hot topic in postcolonial and cultural studies (Thomas 2000; Roque 2010).

This episode should therefore give us pause for thought and provide an opportunity to survey the field. Although whole body donation rates remain low worldwide (McHanwell *et al*. 2008), the international success of Gunther von Hagens’ ‘*Body Worlds*’ (see Von Hagens and Whalley 2002) exhibitions attests to the desire that thousands of people continue to have to be made ‘useful’ after death, even if this use-value involves some of the same elements of mobility and display that are attached to Parker’s skull (for a discussion on the ethics of displaying the dead see Alberti, Bienkowski, and Chapman 2009). At the same time organ donation is increasing in the UK. According to National Health Service (NHS) statistics, the number of living donors increased by 4% in 2012–2013, while the number of donors after brain death grew by 16% in the period 2008–2012 (NHS Blood and Transplant 2013: 5). It is clear, then, that many people recognise the medical value that their bodies and the bodies of their loved ones have, and are concerned about what happens to human remains after death. Scandals and stories of malpractice threaten to unsettle the tenuous social contract we have with those in authority who receive our remains after death.

Newspaper reports were recently circulated about hundreds of rotting corpses that were found abandoned in the basement of Madrid’s Complutense University (The Telegraph 2014). A similar thing happened at the University of Cologne in 2012 (The Guardian 2012). Closer to home, details of the organ retention scandal at Alder Hey Children’s Hospital in Liverpool, which first became public in 1999, still remain profoundly shocking and have affected the way thousands of British families engage with the NHS. Meanwhile, in the developing world, illegal organ theft as well the ‘voluntary’ sale of body parts in times of need reveals the market values that never cease to accumulate around human bodies. When people are seen as commodities it is sometimes hard to make distinctions between body parts as trophies that titillate, specimens that educate, or organs that can heal those lucky enough to afford them. As Scheper-Hughes (2001: 26) put it, ‘There is in this process a potential violation to personal identity and, in the use of the parts of someone who has died, to personal memories’. The situation becomes even murkier when artists use body parts and anatomical specimens without consent (Clark 2013).

Perhaps the most disturbing thing about this episode is how historic body parts like Parker’s skull have come to enter the realm of commodity exchange. Although there is nothing new here – the body parts of saints, heroes, and criminals have been stolen, sold, and exchanged for thousands of years – we should still ask: Who decides their value in monetary terms? How much does ‘authenticity’ really cost? And should owners of relics which have been collected through violence and trauma really have the right to sell them on at a profit to another private buyer?

In June 2014 the skull of a Civil War soldier found at Gettysburg was listed for auction in Pennsylvania with a guide price of US$50,000. It was only after a public outcry and protests from the National Park Service that the skull was withdrawn from auction. Instead, it was donated to the Gettysburg Foundation, which plans to bury it with military honours once its authenticity is confirmed. For those who opposed the sale, it was clear that a public auction was no way to treat the remains of a soldier who had lost his life on the battlefield, a ‘sacred burial ground’ in the words of an official at the Gettysburg National Military Park (Reuters 2014).

Stories like this are unsettling because of the continuities between the merchandising of human remains in the past and the merchandising of organs, blood, and tissue in the present – continuities made clear in scholarly literature (Richardson 2006; MacDonald 2005: 186–189) and museum exhibitions (for example ‘*Doctors, Dissection, and Resurrection Men*’ at the Museum of London in 2012–2013). Fragments like Parker’s skull must be part of a debate which purposefully sets out to further unsettle the ever-tenuous contracts between the living and the dead. This can be done by cultivating an intimate engagement with body parts that are on display or advertised for sale, an engagement which is, by necessity, disturbing and never conclusive (Parry 2008).

We start by being concerned when body parts, no matter how old or historic they are, are put up for sale. We need to ask questions about where the human remains have come from, where they are going, and about the appropriateness of display. The ethical imperative to historicize items for sale increases in importance when the body part has come from a criminal who has been executed. In the absence of consent, and the violence implicit in the provenance of the body part, context becomes a crucial means of informing the public about what they are seeing in a museum or bidding on in an auction house.

The key selling point of Parker’s skull seemed to be its criminality. While most cultures have an urge to see criminals suffer and die in public, it must be remembered that this attribution of criminality is historically contingent and never stable. Think of Jesus Christ, executed as a criminal and now considered the central figure in Christianity; or Oliver Cromwell, buried in 1658 with the highest honours that could be bestowed, but exhumed and hung as a regicide and criminal under the restored monarchy just a few years later; or the various political corpses restored to national honour in the post-socialist states of Eastern Europe (Verdery 1999). Is Parker’s skull, as that of a Wiltshire thief, too banal to draw forth the kind of ethical debate that might emerge if the auctioneer sold the skull of a Jewish victim of Nazi genocide? What if he had sold the skull of one of the more than 50 men hanged for sodomy in Britain between 1800 and 1835 (Harvey 1978)? It is probable that such a sale would not be considered wacky at all, but something replete with trauma and disgust. The key thing here is that context can bolster personhood. We cannot but help feel the skull of an executed Jew, a homosexual, or a Gettysburg soldier retains its subjectivity, an aura of presence that resists objectification as a commodity, because of the proximate histories and deeply felt debates that encrust such skulls. On the other hand, one might say that Parker is just an ordinary criminal nobody, and his skull a silent and ahistorical object that would fit nicely on a mantelpiece.

## Museums and the Dead as ‘Subject-Objects’

This sale also raises the question of how curators can deal with the inscription of criminality on flesh and bone – literally in this case. Around the time that Parker was executed, the contested science of phrenology was gaining adherents among medical professionals and collectors in Britain and North America. Phrenologists believed that a person’s characteristics could be determined by particular ‘organs’ in the brain that could be felt on the skull. Phrenologists were fascinated by the skulls of notorious criminals, and were frequently to be found amid the public scrum at a dissection, bribing or cajoling their way into the room to make their observations. Cranial measurements were then used to demonstrate that executed criminals had demonstrable tendencies – towards ‘destructiveness’ or ‘acquisitiveness’ for example. Labelling body parts as criminal has a long and deleterious past, but we should not simply denounce all collection and display of skulls without consent as an inherently bad thing.

Museums in the twenty-first century, many of which display human remains gathered without consent in the nineteenth century, are now animating the subjects behind the glass, positing them as lively subject-objects (Alberti and Hallam eds. 2013). In ‘*Narrative Remains*’, a 2009 art project at the Hunterian Museum in London, Karen Ingham gave voice to some of the dead specimens from the private collection of John Hunter (a renowned Scottish surgeon; 1728–1793) (Ingham 2009). Another approach, which leads with empathy and context, is to encourage the public to donate money to maintain and restore body parts on display. For instance, in 2013 the Mütter Museum in Philadelphia initiated a ‘*Save our Skulls*’ campaign to encourage people to ‘adopt’ one of the 139 skulls sold to the museum in 1874 by the Viennese physician Josef Hyrtl. This succeeded in raising funds for the museum, but also allowed members of the public to cultivate intimate attachments to ‘their’ skulls, based around repeat visits. Hyrtl, incidentally, was billed as someone who sought to disprove the theories of phrenologists by collecting the skulls of Caucasians, some of whom happened to have been executed as criminals (Time 2013). Franz Boas is another example of a physical anthropologist that collected skulls in order to bolster anti-racist evolutionary approaches (on his ethical utilitarianism, see Pöhl 2008). Given a thoughtful curatorial approach such as this, museum objects can simultaneously become subjects that are, in the words on the banner outside the Mütter Museum, ‘disturbingly informative’.

These kinds of interventions are occurring at a time when the legal landscape around the procurement and storage of dead bodies is changing. In the USA the Native American Graves Protection and Repatriation Act (1990) has transformed the way cultural institutions interact with indigenous peoples and their own curatorial legacies (Quigley 2001: 205–23). In the UK the Human Tissue Act 2004 and the Human Tissue (Scotland) Act 2006 have enshrined the consent principle and criminalised DNA theft. Although in this post-Alder Hey context many museums and anatomical institutions are now open to a collective conversation on the origins and framing of their organic material, there is still a huge diversity of opinion and policy on what to do with human remains. For instance, UK museums and universities hold the world’s largest collection of Aboriginal bodies and body parts, and for decades stakeholders have been calling for their repatriation to Australia. This campaign received international attention in 2010 when Prince William offered his support in the search for the skull of the resistance leader Pemulwuy, sent to Sir Joseph Banks in London in the early nineteenth century (Herald Sun 2012). Although the Natural History Museum in London has been unable to locate this skull, in 2011 it returned the remains of over 130 Torres Strait Islanders to their communities of origin (Australian Geographic 2011).

Yet even within the same museum there are ongoing debates which make any one policy on the repatriation or reburial of human remains extremely problematic. In 2004 the skeleton of William Corder, infamous for the murder of Maria Martin in the Red Barn in 1827, was released from the Hunterian Museum in London following lobbying from a descendant. After a religious service, the skeleton was cremated and buried in Corder’s home village of Polstead, Suffolk (Corder’s scalp and a book bound in his skin remain on display at the Moyse’s Hall Museum in Bury St Edmunds (McCorristine 2014)). However, in contrast to this, the skeleton of Charles Byrne, the 7ft 7in tall ‘Irish Giant’, is still on display, despite evidence that he requested a burial at sea in a lead coffin in order to evade the hands of John Hunter (Doyal and Muinzer 2011). This case has generated important discussions about consent and the morality of displaying the dead against their will. Although the Human Tissue Act cannot be applied retrospectively to Byrne, the Director of the Hunterian has argued that the educational and medical benefits of keeping Byrne in the museum outweigh the case for his burial (The Guardian 2011). It is clear that for museums there is a balance to be struck between correcting historical wrongs and displaying inherited (and charismatic) human remains in a manner appropriate to audiences who want to see them. One way of doing this might be to harness the ethical and emotional response to Byrne’s skeleton and make this part of the display, using his subjectivity to voice the issues of context and consent that must be central to medical museums in the twenty-first century. As one group of scholars put it, ‘Hunter’s actions were immoral by modern standards, but apologising for the deeds of others long dead just salves the consciences of the living and has no effect on the deceased. We cannot change the past, but we can learn from it’ (Smith *et al*. 2012: e556).

Museums might look to changes in the attitudes of anatomists in this regard. Although historically laws have not prevented the unethical treatment of dead bodies in medical institutions (see above), there are positive signs that anatomists worldwide have recognized the potentially traumatic impact of their work. For instance, annual commemorative and burial services are now held by most anatomical institutes, at which family and friends of donors, as well as anatomists and students, gather to remember donors. There have also been attempts in the Virginia General Assembly to publicly commemorate the contributions of illicitly obtained African American corpses to medical science (The Commonwealth Times 2014). Whether donated with consent, or taken without consent, body parts, in this new understanding, are seen by some as gifts with values that lie outside of the market economy.

## Conclusion

It is high time that the kind of ethical reflections that are taking place in museums and anatomical institutes reach auction houses. Ebay forbids the sale of body parts through its website but they continue to be bought and sold in prestigious auction houses like Christies. Being dry and stable human remains, skulls are notoriously mobile and can be stored indefinitely and cheaply. Cases similar to this will undoubtedly happen again, but they should always be contentious. Thinking of Parker’s skull as Lot Number 202 reproduces the criminality that was stamped on his body after being hanged in 1813. Is this an antique piece or a part of a person who did not consent to being sold to a private buyer? While it might be easy for the successful bidder to think of the skull as a curious object, it is up to others to point out the criminality that led to its objectification and sale in the first place. Taking onboard the more challenging idea that body parts are ‘polysemic’ and always retain some forms of objectification (Alberti 2011: 8) means that displaying Parker’s skull in a museum, with context provided and intimacy or adoption encouraged, is something very different than putting it on a mantelpiece at home (for an overview of museum exhibition ethics see Gazi 2014). In both cases the skull is a lively subject-object, but the public understanding remit of not-for-profit museums demands that they ask visitors to think about where it came from, why it ended up here, and where or whether it might move next (see the comments of Sharon Ament^2^, The Independent 2012). These kinds of discussions are not possible when a skull is purchased by a private bidder, and so Parker’s voice will remain as silent as poor Yorick.

Given the current bioethical debates surrounding the Human Tissue Act, the *Body Worlds* exhibitions, and organ trafficking worldwide, museums that continue to display human remains should be disturbingly informative by raising issues of context and consent. However, the auction of Parker’s skull did not raise these issues at all, and by making a profit from selling human remains to a private bidder, it is a reminder of the dark value of criminal bodies in the global marketplace of body parts. The sale was simply disturbing in the wrong way.

## Notes

2 In 2012 it was reported that Sharon Ament, the Director of the Museum of London, was so affected by the exhibition ‘*Doctors, Dissection and Resurrection*’, that she decided to leave her body to science after death. Ament said the exhibition ‘leaves you in no doubt that when it comes to going under the surgeon’s knife we are better off today…It’s a very reflective exhibition, it is not sensationalist. People may have strong reactions, but a museum should try and elicit strong reactions from their visitors’.
